# Dysregulation of the Immune Microenvironment Contributes to Malignant Progression and Has Prognostic Value in Bladder Cancer

**DOI:** 10.3389/fonc.2020.542492

**Published:** 2020-12-16

**Authors:** Zongtai Zheng, Shiyu Mao, Wentao Zhang, Ji Liu, Cheng Li, Ruiliang Wang, Xudong Yao

**Affiliations:** Department of Urology, Shanghai Tenth People′s Hospital, Tongji University School of Medicine, Shanghai, China

**Keywords:** bladder cancer, Cell Type Identification by Estimating Relative Subsets of RNA Transcripts, immune, nomogram, prognostic signature

## Abstract

**Objective:**

The malignant progression from non-muscle-invasive bladder cancer (NMIBC) to muscle-invasive bladder cancer (MIBC) is common and has detrimental effect on patients. We aimed to elucidate the underlying mechanisms of the malignant progression from an immunological perspective and establish a reliable signature for prognostic prediction and immunotherapeutic strategies.

**Methods:**

The Cell Type Identification by Estimating Relative Subsets of RNA Transcripts algorithm was applied to the GSE32894 data set to identify the different tumor-infiltrating immune cells involved in NMIBC and MIBC. Using weighted gene correlation network analysis, survival analysis and least absolute shrinkage and selection operator Cox analysis, we established an immune prognostic signature (IPS) based on 14 overall survival-associated immune genes in The Cancer Genome Atlas (TCGA). Functional enrichment analyses and nomogram were performed to explore the potential effects and prognostic performance of the IPS. Furthermore, the RNA-sequence data from our center were used to validate the expression levels of the selected immune genes in BLCA samples.

**Results:**

Diverse proportions of macrophage subtypes were observed between NMIBC and MIBC. Patients with high risk scores had a worse prognosis than patients with low risk scores in training (TCGA) and validation data sets (GSE32894, GSE13507, and GSE48277). The IPS was a useful prognostic factor for patients treated with immunotherapy in the IMvigor210 trial. Hallmarks of multiple oncogenic pathways were significantly enriched in the high risk group. A novel nomogram model was established for prognostic predictions. The dysregulated expression of the selected immune genes between NMIBC and MIBC was also validated in BLCA samples.

**Conclusion:**

Dysregulation of the immune microenvironment promoted the malignant progression from NMIBC to MIBC. The IPS can stratify patients into different risk groups with distinct prognoses and immunotherapeutic susceptibility, thus facilitating personalized immunotherapy.

## Introduction

Bladder cancer (BLCA) ranks as the 10th most common cancer with a high risk of recurrence (1). The incidence and mortality rates of BLCA in men are nearly four times those in women, making BLCA the sixth most common form of cancer among men (1). According to the degree of tumor invasion, BLCA is classified as either non-muscle-invasive bladder cancer (NMIBC) or muscle-invasive bladder cancer (MIBC). About 75% of newly diagnosed BLCA patients have NMIBC while the remaining patients have MIBC (2).

The treatment and prognosis for NMIBC and MIBC are very different. NMIBC patients are usually managed with complete resection of the tumor. If the resected tumor is high-grade T1 or carcinoma in situ, treatment entails intravesical therapy with Bacillus CalmetteGuérin (BCG). This disease is characterized by low rate of mortality but high rate of recurrence. However, MIBC patients are recommended to receive radical cystectomy with neoadjuvant chemotherapy, but the disease has a high rate of metastasis and mortality (3). Five-year rates of malignant progression from NMIBC to MIBC range from 10% to 30% (4), and this malignant progression is detrimental to patients. Despite optimal treatment, there have been no clinically significant changes in the prognosis of BLCA patients suffering from this malignant progression (5). The clinical heterogeneity of BLCA suggests that potential biological mechanisms underlying NMIBC and MIBC may be distinct. Thus, it is urgent to investigate the mechanistic determinants and molecular differences involved in malignant progression from NMIBC to MIBC, which may reveal effective biomarkers for early diagnosis, outcome prediction and target treatment.

Several studies have reported that the dysfunction of tumor-infiltrating immune cells (TIICs) and immune-related genes is associated with tumor stage, tumor grade and patients’ prognoses in BLCA (6–12), indicating that immune dysregulation may be relevant to malignant progression in this disease. However, the different TIICs involved in NMIBC and MIBC have not been fully identified. Recently, the use of immune checkpoint inhibitors (ICIs) has become an important immunotherapy for BLCA (13). ICIs evoke an anti-tumor immune response through blocking the ligation between inhibitory receptors and their ligands. According to the results of clinical trials, only a fraction of BLCA patients benefit from ICIs (13–15). In addition, the utility of inhibitory receptors as prognostic factor for BLCA patients remains unclear owing to conflicting results (16, 17), and the role of inhibitory receptors expression as a prognostic biomarker for response to ICI has not been determined (18). These findings highlight the importance of establishing a reliable signature to identify subgroups of BLCA patients benefit from ICIs.

To investigate the role of immune regulation in the malignant progression from NMIBC to MIBC, we used the Cell Type Identification by Estimating Relative Subsets of RNA Transcripts (CIBERSORT) algorithm for analysis the expression of genes to evaluate a landscape of 22 TIICs and identify different TIICs between NMIBC and MIBC. The expression of 14 overall survival (OS)-associated immune genes were applied to establish an immune prognostic signature (IPS). Our study also evaluated the prognostic value of the IPS in four data sets and analyzed the potential value of the IPS as an immunotherapy biomarker in the IMvigor210 trial. A reliable predictive nomogram based on the IPS was constructed for prognostic prediction. Furthermore, we used the RNA-sequence data from our center to detect the expression of 14 OS-associated immune genes in NMIBC and MIBC samples.

## Materials and Methods

### Ethics

The studies involving human participants were reviewed and approved by the Ethics Committee of Shanghai Tenth People’s Hospital.

### Data Collection

Forty-nine BLCA samples (35 NMIBC and 14 MIBC) were obtained from patients in Shanghai Tenth People’s Hospital who underwent either transurethral resection of bladder tumor or radical cystectomy between November 2019 and April 2020. Informed consent was prior obtained. Total RNA was extracted from the formalin-fixed paraffin-embedded specimens with the RNeasy FFPE Kit (Qiagen, Hilden, Germany) after deparaffinization with Xylen. Paired-end libraries were synthesized from 100 ng/ml of total RNA using ABclonal Whole RNA-seq Lib Prep kit. Sequence data were obtained using the Illumina NovaSeq 6000 platform.

The microarray data sets GSE32894, GSE13507, GSE48277 and GSE31684 with their corresponding clinicopathological features were obtained from the Gene Expression Omnibus (GEO) database of the NCBI database (https://www.ncbi.nlm.nih.gov/). The mRNA (RNA-sequence) Fragments Per Kilobase of transcript per Million Fragments standardized expression data set and corresponding clinicopathological features were downloaded for 403 BLCA patients with prognostic information from The Cancer Genome Atlas (TCGA) (http://cancergenome.nih.gov/). The microarray data of IMvigor210 trial were obtained from the website http://research‐pub.gene.com/IMvigor210CoreBiologies. IMvigor210 trial included patients with metastatic urothelial cancer treated with atezolizumab (*PD-L1* inhibitor) (19). Patients without clinical response in the IMvigor210 trial were excluded. The main clinicopathological characteristics of these data sets were listed in **Table 1**. A comprehensive immune-related gene list, identified to actively participate in immunological processes, was extracted from the Immunology Database and Analysis Portal database (https://immport.niaid.nih.gov) (20). Because GSE32894 had the maximum number of patients in NMIBC and MIBC subtypes (including 213 NMIBC patients and 93 MIBC patients), GSE32894 was used to verify the different tumor-infiltrating immune cells (TIICs) and differently expressed immune genes between NMIBC and MIBC.

**Table 1:** Information of data sets used in this study.

Characteristic, n (%)		GSE32894 (n = 308)	GSE13507 (n = 165)	GSE31684 (n = 93)	GSE48277 (n = 159)	TCGA (n = 403)
Subtype	NMIBC	213	103	10	10	3
	MIBC	93	62	83	149	400
	Unknown	2	–	–	–	–
Age (years)	≥65	88	69	65	31	254
	<65	220	96	28	42	149
	Unknown	–	–	–	86	–
Gender	Male	80	135	68	59	299
	Female	228	30	25	14	104
	Unknown	–	–	–	–	–
Stage	I	–	103	–	6	2
	II	–	1	–	35	128
	III	–	0	–	81	138
	IV	–	1	–	33	132
	Unknown	–	60	–	4	3
Grade	Low	–	105	6	–	21
	High	–	60	87	–	378
	Grade I	48	–	–	–	–
	Grade II	103	–	–	–	–
	Grade III	154	–	–	–	–
	Unknown	3	–	–	–	4
N stage	N0	49	1	–	110	234
	N1-3	22	104	–	47	128
	Unknown	237	60	–	2	41
M stage	M0	–	1	59	157	196
	M1	–	104	34	2	11
	Unknown	–	60	–	–	196

NMIBC, non-muscle-invasive bladder cancer; MIBC, muscle-invasive bladder cancer; TCGA, The Cancer Genome Atlas.

### Quantification of TIICs

CIBERSORT algorithm (https://cibersort.stanford.edu) is a gene-based deconvolution algorithm that uses expression levels of marker genes to quantify the relative scores for 22 TIICs. CIBERSORT *P* value was derived for the deconvolution of each sample using Monte Carlo sampling. The proportions of TIICs in NMIBC and MIBC samples were determined by CIBERSORT at *P*<0.05.

### Weighted Gene Co-Expression Network Approach (WGCNA)

A gene co-expression network was constructed using the “WGCNA” R package v1.68 based on the expression data from the top 40% most variant genes determined by variance analysis (7453 genes) in GSE32894. Firstly, a gene co-expression resemblance measure was used to construct a Pearson’s correlation coefficient matrix for all pairwise genes, and an appropriate β value was determined when the degree of independence (R^2^) was 0.9 to build a scale-free network. Subsequently, a weighted adjacency matrix was transformed into a topological overlap matrix (TOM) that measures the network connectivity of a gene. We used a minimum module size of 100 for the genes dendrogram and some highly similar modules with the correlation higher than 0.8 were merged together. Finally, based on the TOM-based dissimilarity measure, the genes with similar expression profiles were classified into gene modules through average linkage hierarchical clustering. All genes were represented by the expression of module eigengene (ME) in a given module. Modules highly correlated with the NMIBC/MIBC subtype (|r| ≥0.3) were selected for further analyses.

### OS-Associated Immune Genes

Gene Set Cancer Analysis (http://bioinfo.life.hust.edu.cn/web/GSCALite/) was applied to analyze and visualize the pathway activity of OS-associated immune genes in BLCA. Pathway activity module showed the correlation of gene expressions with pathway activity groups (activation and inhibition) that defined by pathway activity score (PAS) (22). Gene Set Enrichment Analysis (GSEA) (http://www.broadinstitute.org/gsea/index.jsp) was conducted to investigate different functions between high and low risk groups. A nominal *P*<0.05 and a false discovery rate <0.25 were considered significant.

### Nomogram Development and Validation

Univariate and multivariate Cox regression analyses were performed to identify the prognostic value of the IPS and other clinicopathological features. Factors significant in multivariate Cox regression analyses were then introduced to establish prognostic nomograms validated by the concordance index (C-index) and calibration plots using the “Rms” R package v5.1 (https://cran.r-project.org/web/packages/rms/index.html) in R. Decision curve analysis (DCA) was employed to compare the net benefits of nomogram and other crucial prognostic factors.

### Statistical Analysis

The differences in the immune cells proportions and the expression levels of 14 immune genes between NMIBC and MIBC were compared using Wilcoxon test. The one-way ANOVA or t-test was used to evaluate risk scores in patients grouped by clinical characteristics. The Kaplan-Meier and log-rank tests were used to compare the OS and disease-free survival (DFS) of the BLCA patients between low and high risk groups. Receiver operating characteristic (ROC) curves were applied to investigate the prediction accuracy of the risk score for predicting prognosis. Principal components analysis (PCA) was carried out to study gene expression patterns in different BLCA subgroups. We used “Pheatmap” R package v1.0.12 and “corrplot” R package v0.84 to generate heatmap and correlation matrix, respectively. To conduct statistical analyses for our study, SPSS 22.0 (SPSS, Armonk, NY, USA), Graphpad Prism V7 (GraphPad Software, Inc.) and R v3.6.1 (https://www.r-project.org/) were employed. A two-sided *P* value<0.05 was considered significant.

## Results

### Evaluation of TIICs

**Figure 1:**
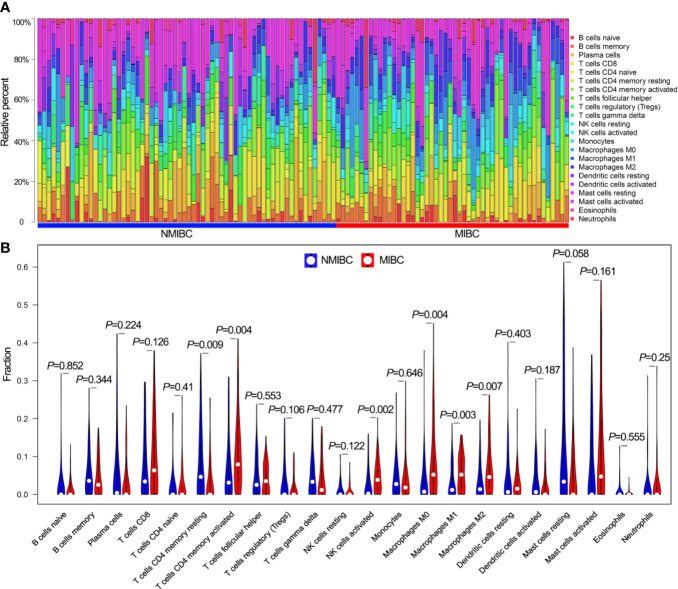
The landscape of 22 TIICs. **(A)** The proportions of 22 TIICs in each sample quantified by CIBERSORT algorithm. **(B)** The difference of the proportions of 22 TIICs between NMIBC and MIBC. TIICs, tumor-infiltrating immune cells; CIBERSORT, Cell Type Identification by Estimating Relative Subsets of RNA Transcripts; NMIBC, non-muscle-invasive bladder cancer; MIBC, muscle-invasive bladder cancer.

The proportions of 22 TIICs in 114 patients (including 64 NMIBC patients and 50 MIBC patients) with a CIBERSORT *P* value <0.05 were quantified (**Figure 1A**). The fractions of memory activated CD4^+^ T cells, activated NK cells and macrophages M0/M1/M2 were consistently higher in MIBC than in NMIBC, whereas the fraction of resting memory CD4^+^ T cells was lower in MIBC (**Figure 1B**).

### Identification of Immune Genes

**Figure 2:**
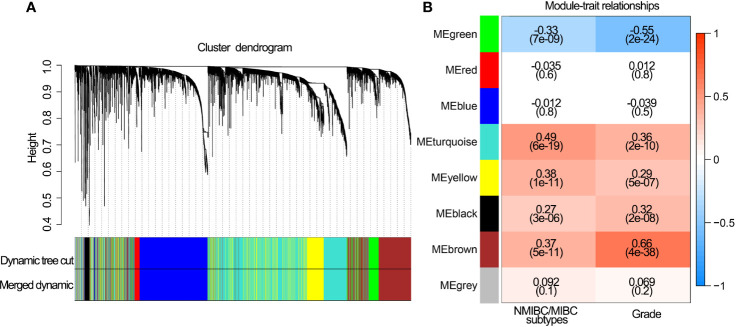
Network construction of the WGCNA and their associations with clinical traits. **(A)** Clustering dendrogram of 292 samples and associated clinical traits. **(B)** The scale-free fit index for various soft-thresholding powers.

The coexpression analysis included 306 BLCA samples with intact clinical information in the GSE32894 data set. After quality check, 292 BLCA samples were selected for subsequent analysis (**Supplementary Figure 1A**). Following WGCNA methodology, β=4 (scale free R^2 =^ 0.91) was regarded as the optimal soft-thresholding value (**Supplementary Figures 1B, C**), and seven modules were identified (**Figure 2A**). The green (r = −0.33, *P*<0.001), turquoise (r = 0.49, *P*<0.001), yellow (r = 0.38, *P*<0.001) and brown modules (r = 0.37, *P*<0.001) showed a high correlation with NMIBC/MIBC subtypes (**Figure 2B**) and thus were regarded as important modules. In these important modules, 592 immune genes (green module, 19 genes; turquoise, 419 genes; yellow module, 80 genes; brown module, 74 genes) were extracted. Univariate Cox regression analysis revealed that 233 of these 592 immune genes were associated with OS in GSE32894 data set. Using cross validation with TCGA data set, 28 immune genes were identified *via* univariate Cox regression analysis and Kaplan-Meier analysis.

### Construction and Performance of IPS

**Figure 3:**
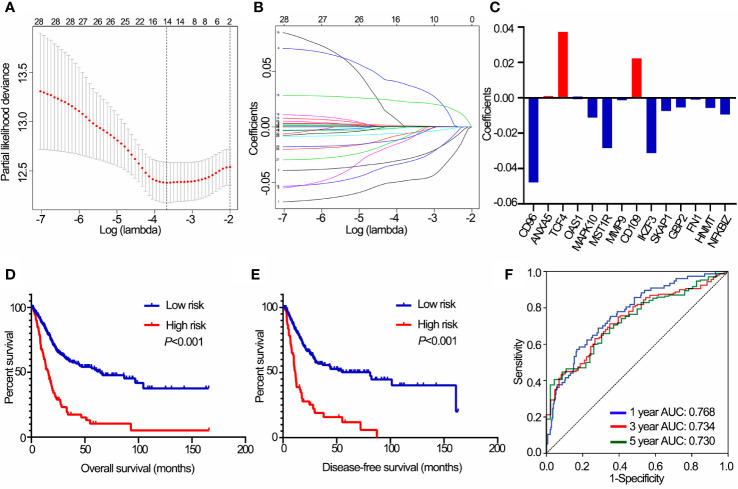
Construction of the IPS using LASSO Cox analysis in TCGA data set. **(A)** Plots of the cross-validation error rates. **(B)** Distribution of LASSO coefficients of OS-associated immune genes. **(C)** Coefficient values of each of the 14 OS-associated immune genes. **(D)** Kaplan-Meier curves of OS for BLCA patients based on the IPS. **(E)** Kaplan-Meier curves of DFS for BLCA patients based on the IPS. **(F)** Receiver operating characteristic curve showed the predictive efficiency of the IPS on 1-, 3-, and 5-year survival rates. IPS, immune prognostic signature; LASSO, least absolute shrinkage and selection operator; TCGA, The Cancer Genome Atlas; BLCA, bladder cancer; OS, overall survival; DFS, disease-free survival. AUC, area under curve.

A total of 28 immune genes were subjected to LASSO Cox regression to construct an IPS for TCGA cohort. As a result, 14 OS-associated immune genes and their corresponding coefficients were determined (**Figures 3A–C**). The risk score of each BLCA patient was calculated using these 14 genes and the formula mentioned above. In TCGA cohort, BLCA patients with high risk scores had shorter OS (*P*<0.001, **Figure 3D**) and DFS (*P*<0.001, **Figure 3E**) than those with low risk scores. ROC curve analyses of IPS showed a high accuracy for survival prediction in TCGA data set (**Figure 3F**).

**Figure 4:**
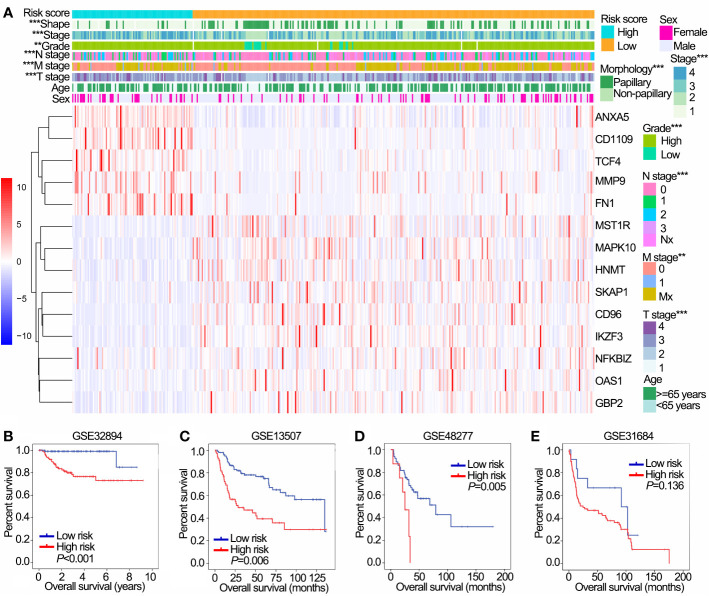
Prognostic value of the IPS in training (TCGA) and validation (GSE32894, GSE13507, GSE48277 and GSE31684) data sets. **(A)** The heatmap shows the expression levels of 14 OS-associated immune genes in low and high risk groups in TCGA data sets. The distribution of clinicopathological features were compared between low and high risk groups. **(B–E)** Kaplan-Meier OS curves for patients assigned to high and low risk groups in GSE32894 **(B)**, GSE13507 **(C)**, GSE48277 **(D)** and GSE31684 **(E)** data sets. ^**^ *P* < 0.01, ^***^ *P* < 0.001. IPS, immune prognostic signature; TCGA, The Cancer Genome Atlas; OS, overall survival.

The distribution of risk scores, patients’ survival status, and expression of 14 OS-associated immune genes were shown in **Supplementary Figures 2A, B**. In addition, significantly different risk scores were observed between patients stratified by stage, grade, T stage, N stage, M stage and morphology (papillary/non-papillary subtypes) in TCGA cohort (*P*<0.001 of all, **Figure 4A**).

We further validated the prognostic value of IPS in four GEO data sets with prognostic information. Except for GSE31684, patients with high risk had worse OS than those with low risk in GSE32894, GSE13507 and GSE48277 (**Figures 4A–D**). Furthermore, patients in the high risk group had poor OS compared with those in the low risk group in NMIBC and MIBC subgroups in both the GSE32894 (**Supplementary Figures 2C, D** both *P*<0.001) and GSE13507 data sets (**Supplementary Figures 2E, F** *P*=0.022 and 0.009, respectively).

### The Potential Value of the IPS as an Immunotherapy Biomarker

**Figure 5:**
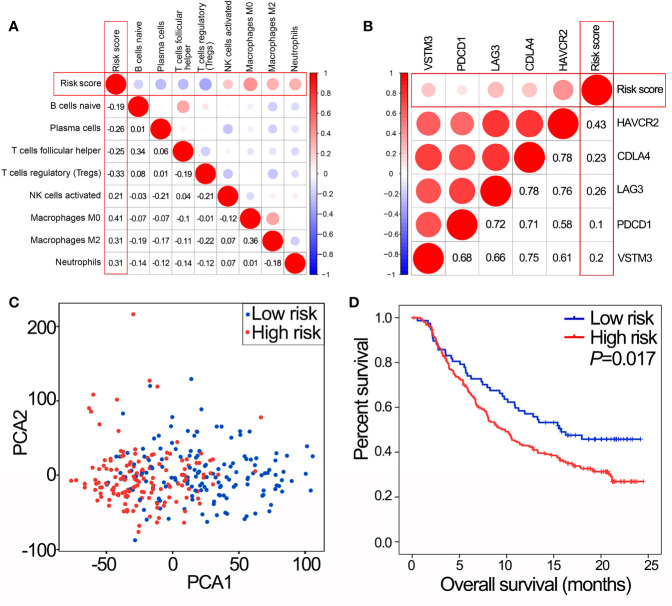
IPS predicts immunotherapy benefits. **(A)** The correlation between risk score and proportions of tumor-infiltrating immune cells in GSE32894 data set. The value represents the value of the correlation coefficient. **(B)** The correlation between risk score and expression of immune checkpoints in GSE32894 data set. The value represents the value of the correlation coefficient. **(C)** PCA of the total RNA expression profile in GSE32894 data set. **(D)** Kaplan-Meier overall survival curves for patients assigned to high and low risk groups in the IMvigor210 trial. IPS, immune prognostic signature; NMIBC, non-muscle-invasive bladder cancer; MIBC, muscle-invasive bladder cancer; PCA, principal components analysis.

IPS was positively correlated with activated NK cells, macrophages M0, macrophages M2 and neutrophils, and negatively correlated with naïve B cells, plasma cells, T regulatory cells (Tregs) and follicular helper T cells (**Figure 5A**, all *P*<0.05). In addition, IPS was positively correlated with the expression of critical immune checkpoint genes (all *P*<0.05, **Figure 5B**). PCA showed a clear distinction between NMIBC/MIBC subgroups in GSE32894 (**Figure 5C**).

In the IMvigor210 trial, patients with high risk scores had poorer OS than those with low risk scores (**Figure 5D**, *P*=0.017). Therefore, the IPS may have the potential clinical value to classify the immunotherapeutic susceptibility for BLCA patients.

### Bioinformatics Analyses of 14 OS-Associated Immune Genes

**Figure 6:**
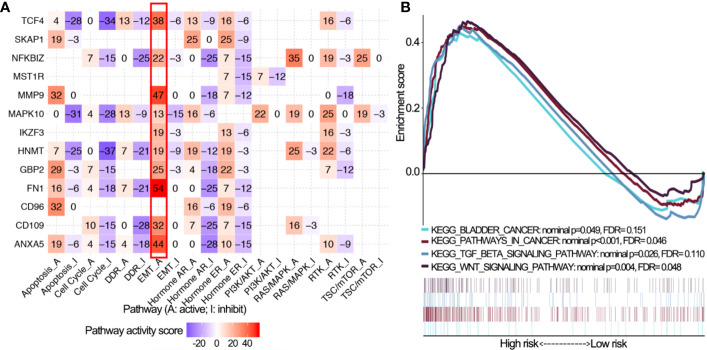
Pathway activity analysis of 14 OS-associated immune genes. **(A)** The correlation of 14 OS-associated immune genes with 10 cancer related pathways (activation and inhibition). The number represents the pathway activity score. **(B)** Gene set enrichment analysis for comparing immune phenotype between high and low risk groups. Significant enrichment of four cancer-related KEGG terms in high risk group. OS, overall survival; KEGG, Kyoto Encyclopedia of Genes and Genomes; FDR, false discovery rate.

Pathway analyses showed that 14 OS-associated immune genes widely associated with most of cancer-related pathways especially epithelial-mesenchymal transition (EMT) and cell cycle (**Figure 6A**). GSEA revealed that malignant hallmarks of tumors, including pathways in cancer, WNT signaling pathway, transforming growth factor-β (TGF-β) signaling pathway and bladder cancer, were mainly enriched in the high risk subgroup (**Figure 6B**).

### Establishment and Validation of Nomograms Based on the IPS

**Figure 7:**
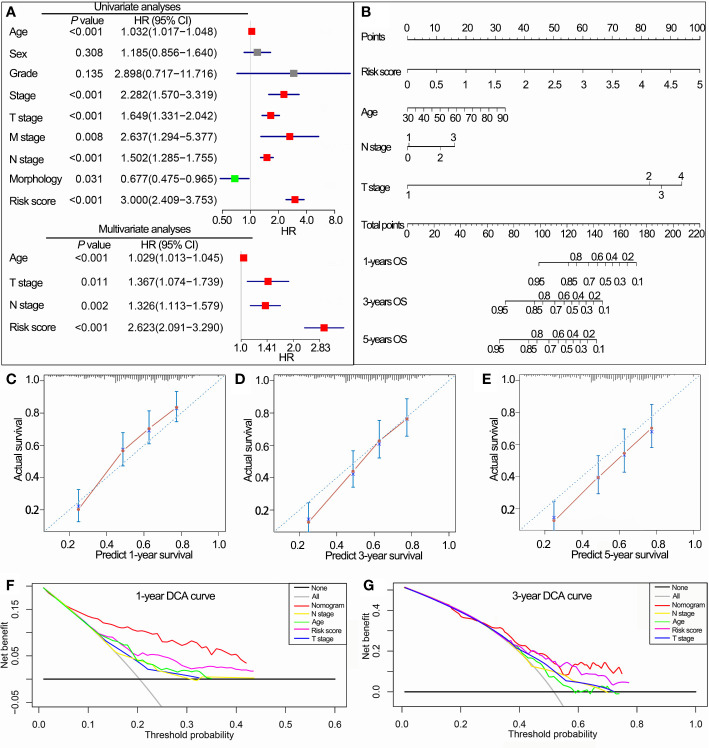
Construction of the nomogram based on the IPS in TCGA data set. **(A)** Univariate and multivariate Cox analyses indicated that the IPS was significantly associated with OS. **(B)** Nomogram for predicting the probability of 1-, 3-, and 5-year OS. **(C–E)** Calibration plots of the nomogram for predicting the probability of 1-, 3-, and 5-year OS. **(F, G)** DCA of the nomogram predicting 1- and 3-year OS. IPS, immune prognostic signature; TCGA, The Cancer Genome Atlas; OS, overall survival; DCA, decision curves analyses; HR, hazard ratio; CI, confidence interval.

In TCGA cohorts, univariate Cox regression analyses demonstrated that IPS, age, stage, grade, T stage, N stage, M stage and morphology, all had prognostic value for OS. These factors were then used for multivariate Cox regression analyses. Consequently, IPS, age, T stage and N stage remained significantly related to OS (**Figure 7A**). Nomograms for 1-, 3- and 5-year OS were established based on IPS, age, T stage and N stage (**Figure 7B**). The C-index of nomograms was 0.73 ± 0.02 (mean ± standard error) for OS, indicating that the prognostic prediction of nomograms was largely consistent with the actual OS in BLCA patients. In addition, calibration plots visually demonstrated that nomogram prediction showed a marked agreement with actual 1-, 3- and 5-year OS (**Figures 7C–E**). DCA revealed that the nomogram showed a larger enhanced clinical net benefit with wider threshold probabilities compared with other crucial prognostic factors (**Figures 7F, G**).

### Validation of 14 OS-Associated mmune Genes

**Figure 8:**
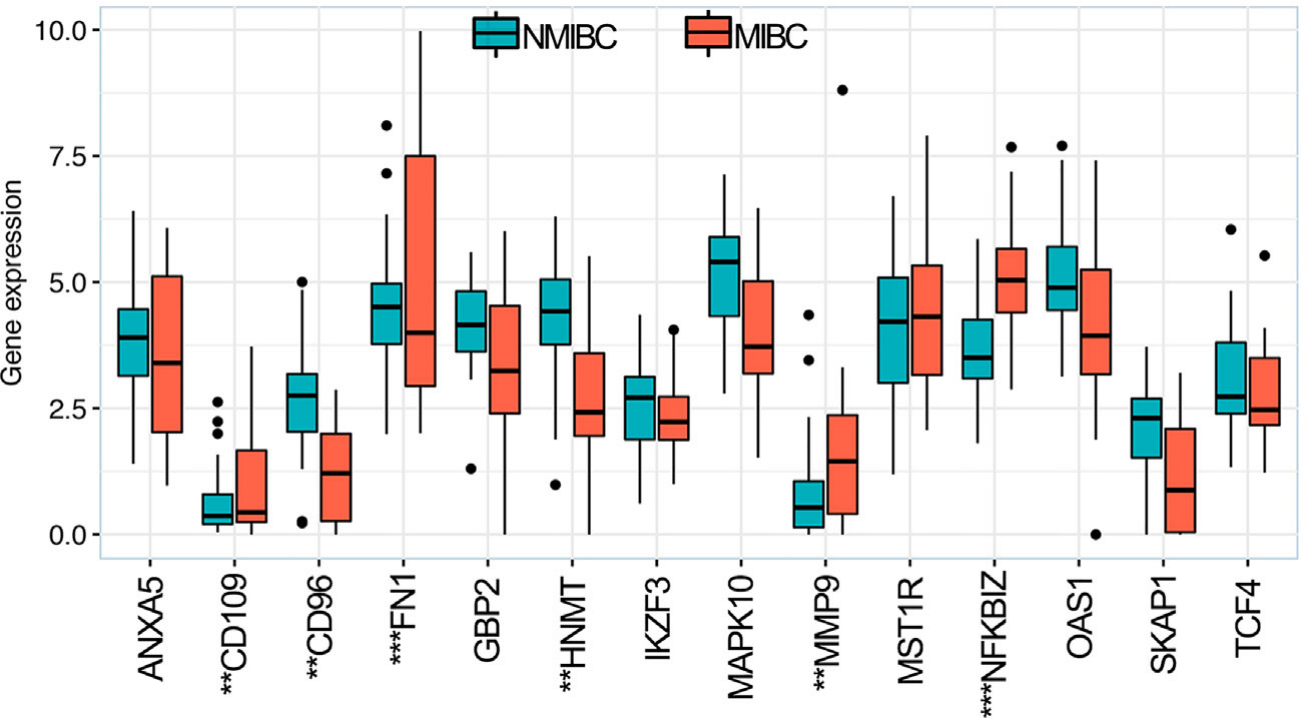
Expression levels of 14 overall survival-associated immune genes in NMIBC and MIBC using the RNA-sequence data of bladder cancer samples in our center. ^**^ *P* < 0.01, ^***^ *P* < 0.001. NMIBC, non-muscle-invasive bladder cancer; MIBC, muscle-invasive bladder cancer.

The RNA-sequence data of BLCA samples from our center were used to validate the expression levels of the 14 OS-associated immune genes in NMIBC and MIBC. The results demonstrated that the expression levels of six genes (CD109, CD96, FNI, HNMT, MMP9 and NFKBIZ) were significantly different between NMIBC and MIBC (**Figure 8**).

## Discussion

Tumor immunology has emerged as a new field which has come of age in the last two decades (10). Tumor immunology contains immune parameters which can provide insights to prognostic predictions and immunotherapeutic strategies in cancers (23, 24), so it is reasonable to revise the current cancer classification system to include these parameters (7, 10). As one of the immune parameters, TIICs reveal the status of the tumor microenvironment (10). In the current study, we used CIBERSOFT to investigate the landscape of TIICs in BLCA and evaluate the different TIICs between NMIBC and MIBC. As key regulators in tumor microenvironments, macrophages perform immunosuppressive functions and promote tumor angiogenesis, progression and metastasis (25–27). It has been reported that BLCA patients with higher proportions of macrophages and macrophages M2 in particular had worse prognoses (10, 28). Our results showed that half of the different TIICs (three of six) between NMIBC and MIBC samples were macrophage subtypes, and the proportions of macrophages M0/M1/M2 were higher in MIBC samples than in NMIBC samples. These findings showed how tumor immunology played an important role in the malignant progression from NMIBC to MIBC, and the diversity of macrophages may enhance malignant progression.

As BLCA is caused by a complex etiology with polygenic involvement rather than a single gene acting in isolation (29, 30), and only a few single molecular biomarkers have been recommended by guidelines or validated for clinical application (31). Thus, we focused on the application of a multiple gene signature (32, 33). Building a risk signature based on the expression levels of immune genes for prognostic predictions and immunotherapeutic strategies is regarded as a vital topic of research (30, 34, 35). Through WGCNA, survival analysis and LASSO Cox regression, we established an IPS based on 14 OS-associated immune genes, which are differentially expressed between NMIBC samples and MIBC samples. The results showed that the IPS achieved good performance in prognostic stratification in training and validation data sets, in which patients in the high risk group showed significantly poorer OS than patients in the low risk group. The IPS also had prognostic value in NMIBC and MIBC subgroups in the GSE32894 and GSE13507 data sets. High risk scores were associated with adverse pathologic and clinical features. PCA revealed that BLCA patients from the low or high risk subgroups were classified in distinct directions indicating differences in the immune phenotype. Taken together, the IPS demonstrated an oncogenic function in malignant progression and was identified as a useful prognostic tool for prognostic prediction in BLCA.

The landscape of TIICS and the expression of critical immune checkpoint genes is of great significance in immunotherapeutic strategies (9, 17, 36, 37). Further analysis was conducted to evaluate correlations of the IPS with TIICs and critical immune checkpoints. We found that the IPS was correlated with eight of 22 TIICs and was especially and positively correlated with macrophage subtypes which perform immunosuppressive functions and have a negative prognostic effect on BLCA. This result confirmed the finding that the IPS was a negative prognostic factor for BLCA patients and that the heterogeneity of TIICs was important in BLCA progression (7, 25, 28). In addition, BLCA patients with high risk scores had higher expression of critical immune checkpoint genes, indicating that immunosuppression partly contributed to the poor prognosis of these patients. In the IMvigor210 trial, we validated the robustness of the IPS and the ability to predict the response to immunotherapy. In this way, the IPS may have the potential to facilitate individualized immunotherapy in BLCA.

Notably, dysregulated expression of 14 OS-associated immune genes was associated with the activity of multiple oncogenic pathways, especially the EMT signaling pathway that is regarded as a trigger for malignant progression and is associated with neoplastic invasion and prognosis in BLCA (33, 38). The presence of EMT-like features was associated with the upregulation of immune-suppressive signals/targets in human cancers, but the directionality of this association and mechanistic determinants are not well understood (39). One study has found that higher expression of EMT-related genes was associated with lower response rates and shorter DFS and OS among metastatic urothelial cancer patients treated with a PD-1 inhibitor (nivolumab) (36) GSEA revealed that the malignant hallmarks of tumors, including pathways in cancer, the WNT signaling pathway, TGF-β signaling pathway and bladder cancer were significantly associated with the high IPS subgroup. All of these findings indicated that the two subgroups identified by IPS were closely correlated with the malignancy of BLCA.

As the IPS could distribute BLCA patients into subsets with different prognoses and immunophenotypes, we integrated the IPS and other independent prognostic factors to establish a novel nomogram for clinical practice. The C-index value revealed that the nomogram was a useful evaluation tool for prognostic predictions. Calibration curves and decision curve analysis confirmed the prognostic significance and predictive superiority of the nomogram model. This nomogram based on the IPS may serve as a useful evaluation tool to perform mortality risk identification in BLCA patients.

The results of RNA-sequence in our center indicated that six (CD109, CD96, FNI, HNMT, MMP9 and NFKBIZ) of the 14 OS-associated immune genes exhibited dysregulated expression between NMIBC and MIBC, indicating that dysregulated expression levels of these genes served an important role in the malignant progression of BLCA. Hagikura et al. (40) reported that the CD109 expression was negatively associated with tumor stage and pathological grade *via* inhibiting TGF-β/Smad signaling in BLCA, which was corresponded with the results of GSEA and RNA-sequence in our center. Wong et al. (41) found that the MMP9 expression was significantly associated with higher pathological grade, higher tumor stage and a shorter OS. However, there is no *in vivo* evidence investigating the role of the rest four OS-associated immune genes in the malignant progression of BLCA.

In summary, our study elucidated the mechanism of the malignant progression from NMIBC to MIBC from an immunological perspective. The diversity of macrophages may enhance malignant progression. The IPS based on 14 OS-associated immune genes may be a clinically promising signature for the prediction of prognosis and immunotherapeutic susceptibility. Biological functional analysis of IPS and the 14 immune genes has thus provided new insights into the malignant progression of BLCA. Dysregulated expression of the OS-associated immune genes was validated in BLCA samples by RNA-sequence in our center. Some of the immune genes should be prioritized for additional functional studies and mechanistic analyses.

## Data Availability Statement

Publicly available datasets were analyzed in this study, these can be found in The Cancer Genome Atlas (http://cancergenome.nih.gov/); the NCBI Gene Expression Omnibus (GSE32894, GSE13507, GSE48277, and GSE31684).

## Ethics Statement

The studies involving human participants were reviewed and approved by The Ethics Committee of Shanghai Tenth People’s Hospital. The patients/participants provided their written informed consent to participate in this study.

## Author Contributions

ZZ and XY conceived and designed the study. XY acquired the funding. ZZ, SM, and WZ collected and collated the data and tumor samples. All the authors were involved in the analysis and interpretation of data. ZZ wrote the manuscript, XY, SM and WZ critically reviewed and revised the manuscript. JL, CL, and RW designed the tables and figures. All authors contributed to the article and approved the submitted version.

## Funding

This study was funded by the Shanghai Science Committee Foundation (grant number 19411967700) and the National Natural Science Foundation of China (grant number 81472389).

## Conflict of Interest

The authors declare that the research was conducted in the absence of any commercial or financial relationships that could be construed as a potential conflict of interest.

## Supplementary Material

Supplementary Figure 1 Network construction of the WGCNA. **(A)** Clustering dendrogram of 292 samples and associated clinical traits. **(B)** The scale-free fit index for various soft-thresholding powers. **(C)** The mean connectivity for various soft-thresholding powers. WGCNA, weighted gene correlation network analysis; NMIBC, non-muscle-invasive bladder cancer; MIBC, muscle-invasive bladder cancer.

Supplementary Figure 2 Prognostic value of the IPS in training (TCGA) and validation (GSE32894 and GSE13507) data sets. **(A)** Risk scores distribution, OS status of each patient, and heatmaps of 14 OS-associated immune genes. **(B)** Risk scores distribution, DFS status of each patient, and heatmaps of 14 OS-associated immune genes. **(C, D)** Kaplan-Meier OS curves for NMIBC patients **(C)** and MIBC patients **(D)** assigned to high and low risk groups in GSE32894 data set. **(E, F)** Kaplan-Meier OS curves for NMIBC patients **(E)** and MIBC patients **(F)** assigned to high and low risk groups in GSE13507 data set. IPS, immune prognostic signature; TCGA, The Cancer Genome Atlas; OS, overall survival; DFS, disease-free survival; NMIBC, non-muscle-invasive bladder cancer; MIBC, muscle-invasive bladder cancer.
