# Supplementary figures and images for: Dysregulation of the Immune Microenvironment Contributes to Malignant Progression and Has Prognostic Value in Bladder Cancer
Source: Front Oncol. 2020 Dec 16;10:542492. doi: 10.3389/fonc.2020.542492 (PMC7773013; doi:10.3389/fonc.2020.542492)

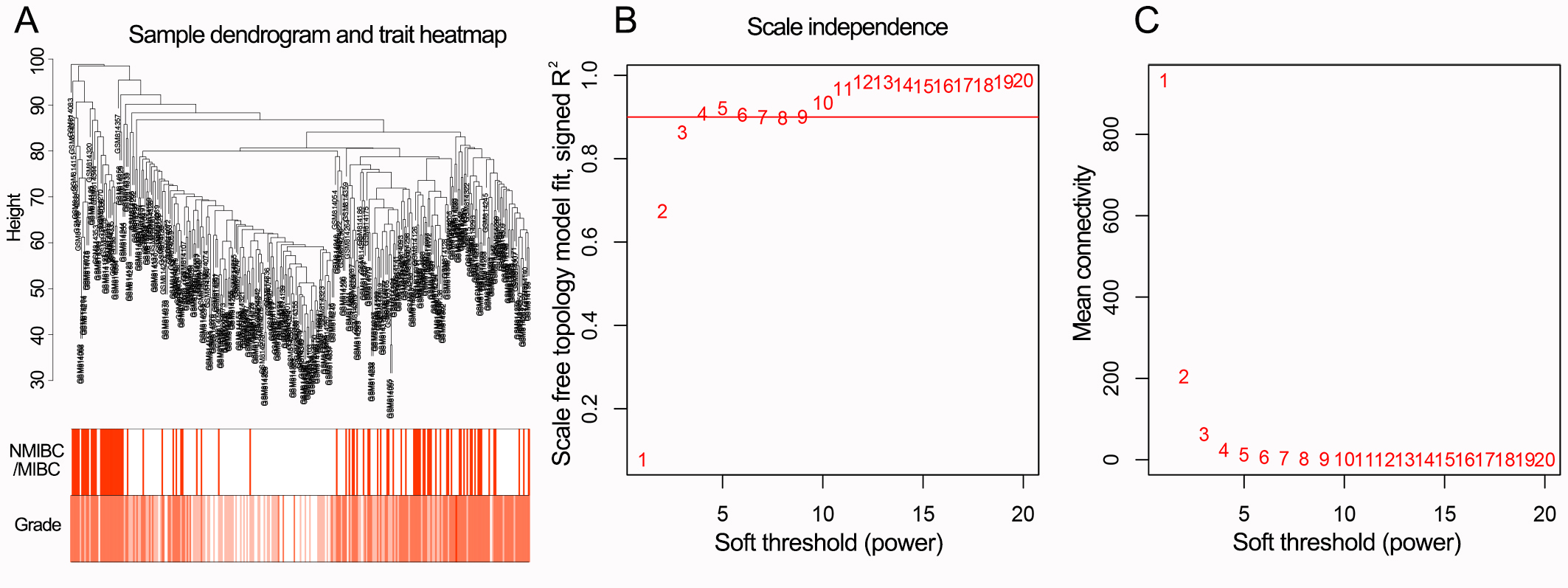

Supplement: Supplementary Figure 1 — Network construction of the WGCNA. (A) Clustering dendrogram of 292 samples and associated clinical traits. (B) The scale-free fit index for various soft-thresholding powers. (C) The mean connectivity for various soft-thresholding powers. WGCNA, weighted gene correlation network analysis; NMIBC, non-muscle-invasive bladder cancer; MIBC, muscle-invasive bladder cancer. [file Image_1.jpeg]

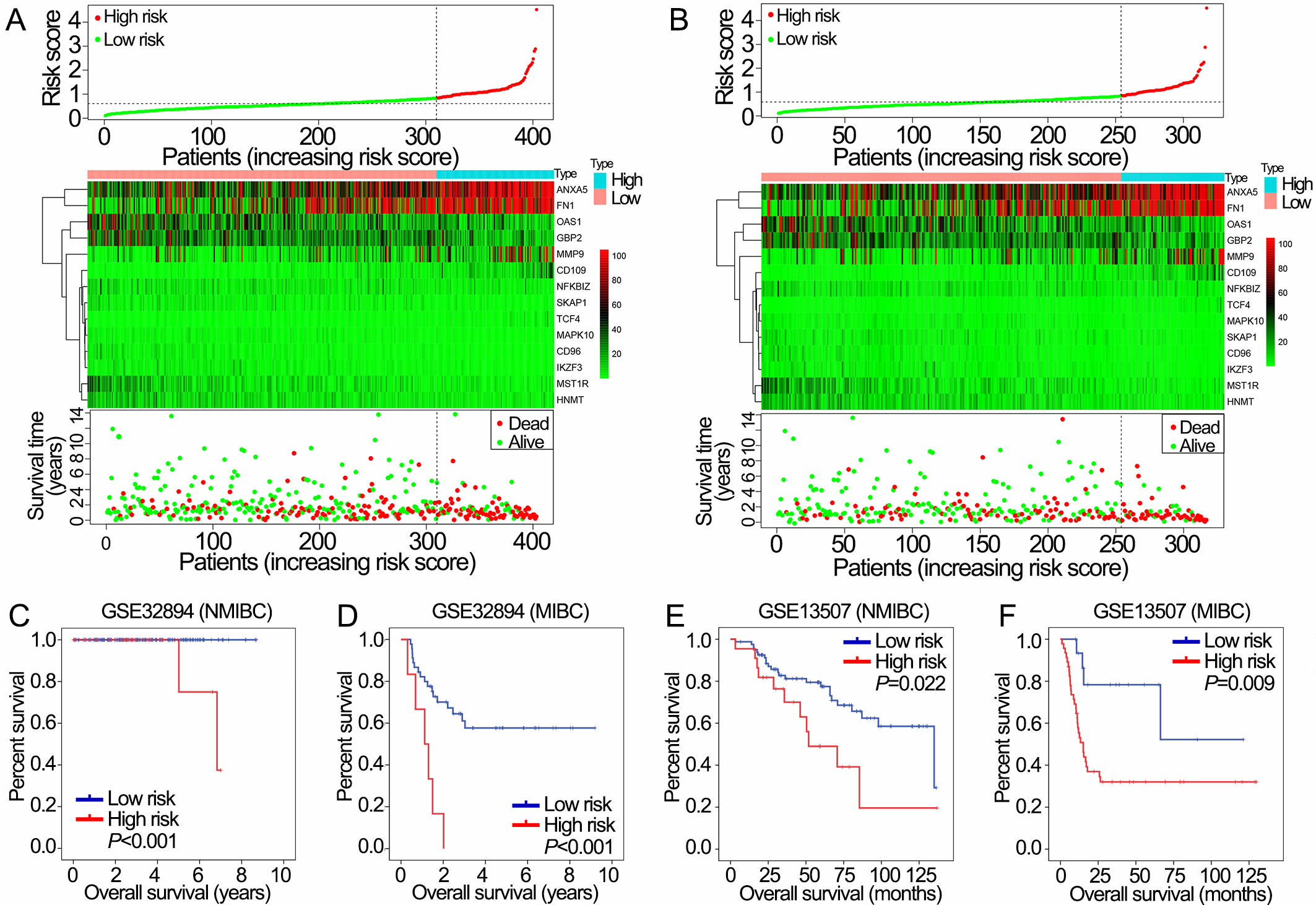

Supplement: Supplementary Figure 2 — Prognostic value of the IPS in training (TCGA) and validation (GSE32894 and GSE13507) data sets. (A) Risk scores distribution, OS status of each patient, and heatmaps of 14 OS-associated immune genes. (B) Risk scores distribution, DFS status of each patient, and heatmaps of 14 OS-associated immune genes. (C, D) Kaplan-Meier OS curves for NMIBC patients (C) and MIBC patients (D) assigned to high and low risk groups in GSE32894 data set. (E, F) Kaplan-Meier OS curves for NMIBC patients (E) and MIBC patients (F) assigned to high and low risk groups in GSE13507 data set. IPS, immune prognostic signature; TCGA, The Cancer Genome Atlas; OS, overall survival; DFS, disease-free survival; NMIBC, non-muscle-invasive bladder cancer; MIBC, muscle-invasive bladder cancer. [file Image_2.jpeg]
